# Whole lung morphometry with 3D multiple b‐value hyperpolarized gas MRI and compressed sensing

**DOI:** 10.1002/mrm.26279

**Published:** 2016-06-10

**Authors:** Ho‐Fung Chan, Neil J. Stewart, Juan Parra‐Robles, Guilhem J. Collier, Jim M. Wild

**Affiliations:** ^1^ POLARIS, Academic Unit of RadiologyUniversity of SheffieldUnited Kingdom; ^2^ Insigneo, Institute for *in silico* medicineSheffieldUnited Kingdom

**Keywords:** hyperpolarized ^3^He, compressed sensing, lung morphometry, stretched exponential model, ADC mapping

## Abstract

**Purpose:**

To demonstrate three‐dimensional (3D) multiple *b*‐value diffusion‐weighted (DW) MRI of hyperpolarized ^3^He gas for whole lung morphometry with compressed sensing (CS).

**Methods:**

A fully‐sampled, two *b*‐value, 3D hyperpolarized ^3^He DW‐MRI dataset was acquired from the lungs of a healthy volunteer and retrospectively undersampled in the *k*_*y*_ and *k*_*z*_ phase‐encoding directions for CS simulations. Optimal k‐space undersampling patterns were determined by minimizing the mean absolute error between reconstructed and fully‐sampled ^3^He apparent diffusion coefficient (ADC) maps. Prospective three‐fold, undersampled, 3D multiple *b*‐value ^3^He DW‐MRI datasets were acquired from five healthy volunteers and one chronic obstructive pulmonary disease (COPD) patient, and the mean values of maps of ADC and mean alveolar dimension (*Lm*_*D*_) were validated against two‐dimensional (2D) and 3D fully‐sampled ^3^He DW‐MRI experiments.

**Results:**

Reconstructed undersampled datasets showed no visual artifacts and good preservation of the main image features and quantitative information. A good agreement between fully‐sampled and prospective undersampled datasets was found, with a mean difference of +3.4% and +5.1% observed in mean global ADC and *Lm*_*D*_ values, respectively. These differences were within the standard deviation range and consistent with values reported from healthy and COPD lungs.

**Conclusions:**

Accelerated CS acquisition has facilitated 3D multiple *b*‐value ^3^He DW‐MRI scans in a single breath‐hold, enabling whole lung morphometry mapping. Magn Reson Med 77:1916–1925, 2017. © 2016 The Authors Magnetic Resonance in Medicine published by Wiley Periodicals, Inc. on behalf of International Society for Magnetic Resonance in Medicine. This is an open access article under the terms of the Creative Commons Attribution License, which permits use, distribution and reproduction in any medium, provided the original work is properly cited.

## INTRODUCTION

Diffusion‐weighted MRI (DW‐MRI) with hyperpolarized (HP) noble gases is sensitive to changes in lung microstructure through the measurement of the apparent diffusion coefficient (ADC) of the gas in the alveoli 1, 2, 3. However, the measured ADC value can be influenced by non‐Gaussian phase behavior of the DW signal of the gas in the lungs, causing non‐mono‐exponential signal attenuation with increasing *b*‐value. This behavior is determined by the specific diffusion regime, which is influenced by several factors including the DW measurement parameters, gas diffusivity, and the complex alveolar structure 4. Various models of gas diffusion in the lungs have been proposed to address this non‐Gaussian signal behavior, and provide estimates of lung alveolar length scales from the HP gas signal. These include cylindrical geometrical models 5, 6, q‐space transforms 7, and more recently, stretched exponential models 8. All of these approaches have a common requirement for the acquisition of multiple *b*‐value DW‐MRI data, to sample the non‐mono‐exponential diffusion signal. However, multiple *b*‐value acquisition in a single breath‐hold requires long scan times, and multi‐slice two‐dimensional (2D) sequences have been used to date, which do not provide whole lung volumetric coverage for lung morphometry.

Previously used 2D ^3^He DW‐MRI sequences permitted acquisition of approximately five slices with four to six *b*‐values in a single breath‐hold (∼15 s) 8, 9. Three‐dimensional (3D) DW‐MRI sequences designed with a similar slice thickness (∼10–15 mm) and the same number of *b*‐values would require an acquisition time of almost 1 min to obtain whole lung coverage images, which is beyond the limits of a tolerable breath‐hold. Acquisition methods such as radial 10, spiral 11, and parallel radiofrequency (RF) encoding 12 have been previously applied to HP ^3^He lung MRI to decrease image acquisition time. However, these techniques require the use of non‐Cartesian gradient trajectories or custom‐built multichannel RF coils. Compressed sensing (CS) presents an alternative acceleration technique that can be used to reduce the total scan time to within the limits of a breath‐hold by exploiting the sparsity (or compressibility) of lung MR images to acquire a randomly undersampled k‐space 13.

The feasibility of acquisition and reconstruction of HP ^3^He lung MR images with CS was first investigated by retrospectively undersampling and reconstructing fully‐sampled 2D and 3D ventilation images 14. These initial results showed that reductions in scan time could be achieved in 2D and 3D ^3^He ventilation imaging without compromising image quality and functional information. In the same work, prospective undersampled 2D ^3^He DW‐MRI data acquired with CS demonstrated preservation of spatial resolution and mean ADC values compared with fully‐sampled data. Further recent studies with HP gases have incorporated CS to acquire ^3^He‐^1^H images in the same breath‐hold 15, 16, multi‐interleaved 2D ^3^He MRI data for functional and structural mapping including ADC, $T_{2}^{*}$, and B_1_ 17, and also to measure gas flow in the upper airways with phase contrast velocimetry 18.

In this work, CS was implemented to reduce scan time and facilitate 3D multiple *b*‐value HP ^3^He DW‐MRI within a single breath‐hold. Simulations were first performed to investigate the feasibility of 3D ^3^He multiple *b*‐value DW‐MRI with CS undersampling, and reconstructed images were evaluated to ensure that quantitative microstructural information was preserved. Prospective 3D ^3^He multiple *b*‐value DW‐MRI data were subsequently acquired in five healthy volunteers and one chronic obstructive pulmonary disease (COPD) patient. The data were used to calculate maps of ADC values (generated from a 2 *b*‐value exponential fit) and mean diffusion length scale (*Lm*_*D*_) estimates (from a multiple *b*‐value stretched exponential treatment), and these were compared with values obtained from fully‐sampled 3D and 2D multiple *b*‐value DW‐MRI.

## THEORY

### Compressed Sensing

CS allows images to be reconstructed from data that do not fulfill the Nyquist sampling criteria. In this work, images were reconstructed by solving Eq. (1) using a nonlinear conjugate gradient descent algorithm with back‐tracking line search 13:

(1) $$\underset{m}{\text{argmin}}\left\{\|F_{u}m-y{\|}_{2}{}^{2}+\lambda_{1}\|\psi m{\|}_{1}+\lambda_{2}TV(m)\right\}$$

where m is the reconstructed image, ψ is the sparsifying transformation, $F_{u}$ is the undersampled Fourier transform, y is the acquired undersampled k‐space data, TV represents the sum of the absolute variations in the image, and $\lambda_{1}\;$ and $\lambda_{2}$ are the penalty weighting parameters that balance data fidelity and artifact reduction. In this work, no sparsifying transformations were used in the CS simulations, because 3D HP ^3^He lung MR images are naturally sparse and reconstructed data from the simulations were found to be equivalently accurate with and without sparsifying transformations.

### Stretched Exponential Model

Within a ^3^He MR lung imaging voxel, the diffusion of gas atoms is restricted by the walls of airways with different sizes and orientations with respect to the diffusion sensitizing gradient 4. These different diffusion regimes result in differences of apparent diffusion rates, which are not fully compensated by motional averaging for the typical diffusion times used for short‐range ^3^He diffusion measurements 19. This heterogeneity of the apparent diffusivity is further increased by localized diffusion effects induced by large gradients 20, as well as effects related to the airway connectivity (eg, branching 19) and background susceptibility gradients 9. Hence, the measured macroscopic voxel signal can be represented as the superposition of signals with different apparent diffusivities *D* using Eq. (2):

(2) $$\frac{S_{b}}{S_{0}}=\overset{D_{0}}{\underset{0}{\int }}p(D)e^{-bD}dD$$

where $S_{0}$ is the signal when b=0, $S_{b}$ is the signal corresponding to a nonzero *b*‐value, and $D_{0}$ is the free diffusion coefficient of ^3^He in air or N_2_. The probability density function *p(D)* for each voxel can be estimated from the diffusion signal using different approaches 21. The approach employed in this work (see Eqs. 37 and 38 in 21) uses the knowledge that the diffusion MR signal decay of ^3^He in lungs can be well described by a stretched exponential function 8 to obtain a numerical expression for *p(D)*. The distribution of diffusion length scales *L_D_ = * (2*D*Δ)^½^ (ie, root mean squared displacements, in which Δ is the diffusion time) associated with the *D* values can then be calculated for each voxel. The *p*(*L*_*D*_) distributions are a measure of the distribution of microscopic dimensions of the airways (ie, the diffusion‐restricting boundaries) contained within a given voxel. These distributions can be used to calculate the mean diffusion length scale (*Lm*_*D*_) for each pixel. *Lm*_*D*_ values can therefore provide quantitative estimates of the mean acinar airway dimensions within a voxel.

## METHODS

### CS Simulations

All CS simulations and subsequent mean absolute error (MAE), ADC, and *Lm*_*D*_ calculations were implemented in‐house using MATLAB (The Mathworks, Natick, Massachusetts) software. All in vivo MRI experiments were performed under the approval of the UK national research ethics committee.

Fully‐sampled 3D DW HP ^3^He lung MR images were acquired from the lungs of a healthy male volunteer (30 years old) in a 22 s breath‐hold on a GE HDx 1.5 Tesla (T) MR scanner (GE Healthcare, Milwaukee, WI) using a 3D spoiled gradient echo (SPGR) sequence based on that described in 22. A flexible quadrature transmit‐receive RF coil (Clinical MR Solutions, Brookfield, Wisconsin), tuned to the Larmor frequency of ^3^He (48.63 MHz), was used with a gas dosage of 300 mL of HP ^3^He (∼25% polarization), mixed with 700 mL of N_2_. The lung inflation level at imaging had a functional residual capacity plus 1 L (FRC+1L). Images were acquired with sequential phase encoding and the following acquisition parameters: 2× DW interleaves (*b* = 0, 1.6 s/cm^2^), 96 × 78 × 24 matrix, field of view (FOV): 40 × 32.5 × 28.8 cm^3^, effective slice thickness: 12 mm, echo time (TE)/repetition time (TR): 4.2/5.7 ms, diffusion time = 1.6 ms (DW gradient strength = 14.1 mT/m, ramp = 0.3 ms, plateau = 1.0 ms), flip angle = 1.5 ° (hard RF pulse of 0.24 ms duration), bandwidth = ±31.25 kHz. The flip angle was selected such that ∼25% of the initial nonrenewable magnetization remained at the end of the acquisition as a result of RF pulse depolarization.

From the fully‐sampled 3D image data, random k‐space undersampling patterns were generated in the two orthogonal phase‐encoding directions for acceleration factors (AF) ranging from 2 to 5. A Monte Carlo–based algorithm was used with variable density to maximize the incoherence. For each AF, four different k‐space sampling patterns were generated with different probability density functions. Each of these patterns was used to retrospectively undersample the full k‐space dataset, and the corresponding images were reconstructed as follows.

Reconstructions based on Eq. (1) were optimized by minimizing the MAE between the fully‐sampled ADC maps and the CS‐reconstructed ADC maps (MAE_ADC_). The quality of each reconstruction was evaluated using both the MAE_ADC_ and the MAE between the original fully‐sampled magnitude image (*b* = 0) and the CS‐reconstructed magnitude image (MAE_MAG_). For the ultimate goal of quantitative lung microstructural analysis, the MAE_MAG_, MAE_ADC_, and ADC maps were evaluated on a pixel‐by‐pixel basis within a region of interest (ROI) representing the lungs. MAE_MAG_ was calculated using Eq. (3) as follows:

(3) $$MAE_{MAG}=\;\frac{\sum_{i=1}^{N}\sum_{j=1}^{M}\left|CS_{i,j}-Full_{i,j}\right|}{N\times M}$$

where $CS_{i,j}$ and $Full_{i,j}$ denote the normalized pixel values in the CS‐reconstructed and original fully‐sampled images, respectively; and N×M is the total number of pixels in the lung ROI. ADC maps were computed using a pixel‐by‐pixel mono‐exponential fit of signal intensities in the two interleaves of the 3D ^3^He DW‐MRI dataset, as in Eq. (4):

(4) $$ADC=\;\frac{\ln(S_{0}/S_{b})}{b}$$

An asymmetric cutoff of negative (ADC < 0) or physically too high (ADC > D_0_ = 0.88 cm^2^/s) values was applied during the creation of the ADC maps. MAE_ADC_ was calculated using a similar approach to MAE_MAG_, as in Eq. (5)

(5) $$MAE_{ADC}=\;\frac{\sum_{i=1}^{N}\sum_{j=1}^{M}\left|CS\;ADC_{i,j}-Full\;ADC_{i,j}\right|}{N\times M}$$

except that here $CS\;ADC_{i,j}$ and $Full\;ADC_{i,j}$ refer to the pixel values of the ADC maps in the CS‐reconstructed and the original fully‐sampled cases, respectively, as calculated from Eq. (4). Whole lung ADC histograms were generated for each AF, and skewness and full width at half maximum (FWHM) values were derived from each ADC histogram. Skewness of ADC values was calculated using Eq. (6),

(6) $$Skew=\frac{E{\left(x-\mu \right)}^{3}}{\sigma^{3}}$$

where μ is the mean of the ADC values ( x), σ is the standard deviation, and E is the expectation operator.

### Prospective CS Acquisition of 3D ^3^He DW‐MRI

Prospective CS datasets with four DW interleaves were acquired in five healthy volunteers and one COPD patient (spirometric forced expiratory volume in 1 s, FEV_1_ = 31.2% predicted) using the optimal undersampling pattern that was derived from CS simulations with AF = 3. Three‐fold undersampling was chosen because it was the highest AF achievable without introducing significant image blurring, and allowed a scan time reduction from 45 to 15 s, which is a tolerable breath‐hold for most clinical subjects. The 3D CS multiple *b*‐value DW‐MRI dataset was acquired with the following imaging parameters: gas dosage: 300 mL ^3^He mixed with 700 mL N_2_, lung inflation state: FRC + 1L, 4 DW interleaves (*b* = 0, 1.6, 4.2, 7.2 s/cm^2^), 96 × 78 × 24 matrix, FOV: 40 × 32.5 × 28.8 cm^3^, effective slice thickness: 12 mm, TE/TR: 4.2/6.0 ms, diffusion time = 1.6 ms (maximum DW gradient strength = 30 mT/m, ramp = 0.3 ms, plateau = 1.0 ms), flip angle = 1.9 ° (hard RF pulse as previously), bandwidth = ±31.25 kHz. ADC maps were calculated from the first two interleaves, whereas *Lm*_*D*_ maps were derived from all four interleaves using the stretched exponential methodology.

### Prospective CS Acquisition Validation

To validate the ADC and *Lm*_*D*_ microstructural measurements derived from prospective 3D CS data, the same five healthy volunteers were imaged with fully‐sampled 3D and 2D ^3^He DW‐MRI using the scan parameters detailed in the “CS Simulations” section and this section, respectively. 3D fully‐sampled data were also acquired from the COPD patient; however, 2D fully‐sampled data were not acquired because of patient time constraints. The selection of *b*‐values used in all scans was consistent and chosen to ensure that one of the diffusion interleaves corresponded to *b* = 1.6 s/cm^2^, the *b*‐value most commonly used for ^3^He ADC calculations in the literature 1, 3, 23, 24.

2D multiple *b*‐value DW‐MRI data were acquired with similar FOV and slice thickness as the corresponding 3D datasets. Six slices were acquired with 12 mm thickness and 12 mm gap, using a gas dosage of 300 mL ^3^He (mixed with 700 mL N_2_) at a lung inflation state of FRC+1L. Additional imaging parameters were as follows: four DW interleaves (*b* = 0, 1.6, 4.2, 7.2 s/cm^2^), 96 × 72 in‐plane matrix, in‐plane FOV: 40 × 30 cm^2^, TE/TR: 4.9/10 ms, diffusion time = 1.6 ms (maximum DW gradient strength = 30 mT/m, ramp = 0.3 ms, plateau = 1.0 ms), flip angle = 5 ° (sinc RF pulse), bandwidth = ±31.25 kHz.

Comparisons of ADC and *Lm*_*D*_ values were made between each corresponding dataset acquired from each subject; ADC values (from a two *b*‐value, 0 and 1.6 s/cm^2^, mono‐exponential fit) were compared between 3D fully‐sampled and 3D CS acquisitions, whereas *Lm*_*D*_ estimates were compared between 2D fully‐sampled and 3D CS acquisitions. To investigate the agreement between the two sets of measurements, scatter and Bland‐Altman plots were constructed to compare the relative difference in ADC and *Lm*_*D*_ values on a slice‐by‐slice level.

## RESULTS

### CS Simulations

**Figure 1:**
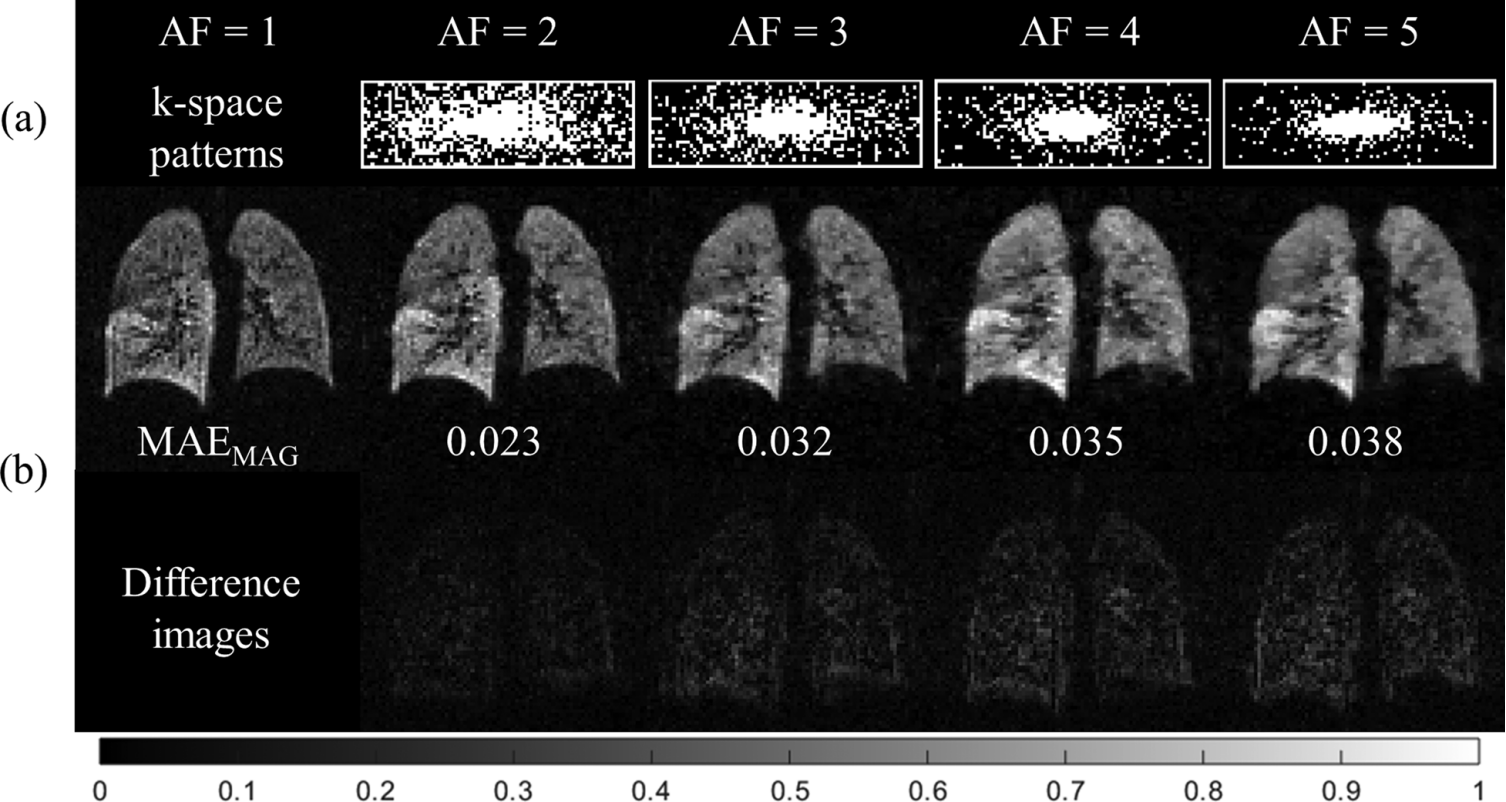
Results from 3D ^3^He CS simulations. (a) Optimal variable‐density k‐space undersampling patterns (78 × 24 pixels) for each AF determined from CS simulations. (b) Example reconstructed magnitude (*b* = 0) and difference images for each AF, with corresponding MAE_MAG_ values.

CS simulations performed on the fully‐sampled two interleaved 3D ^3^He DW‐MRI dataset led to optimal sampling patterns and penalty weight parameters for each AF. The optimal sampling patterns for each AF resulting from the CS simulations are summarized in Figure 1a. An increase in MAE_MAG_ was observed with increasing AF; however, this error (with a maximum value of 3.8% at AF = 5) did not manifest in the appearance of image artifacts. Reconstructed (*b* = 0) images for AFs of 2 and 3 showed good preservation of image details when compared with the fully‐sampled (AF = 1) image (examples shown in Fig. 1b). At AFs of 4 and 5, a loss in image detail was observed as a result of increased blurring resulting from heavier undersampling of high‐frequency k‐space components.

**Figure 2:**
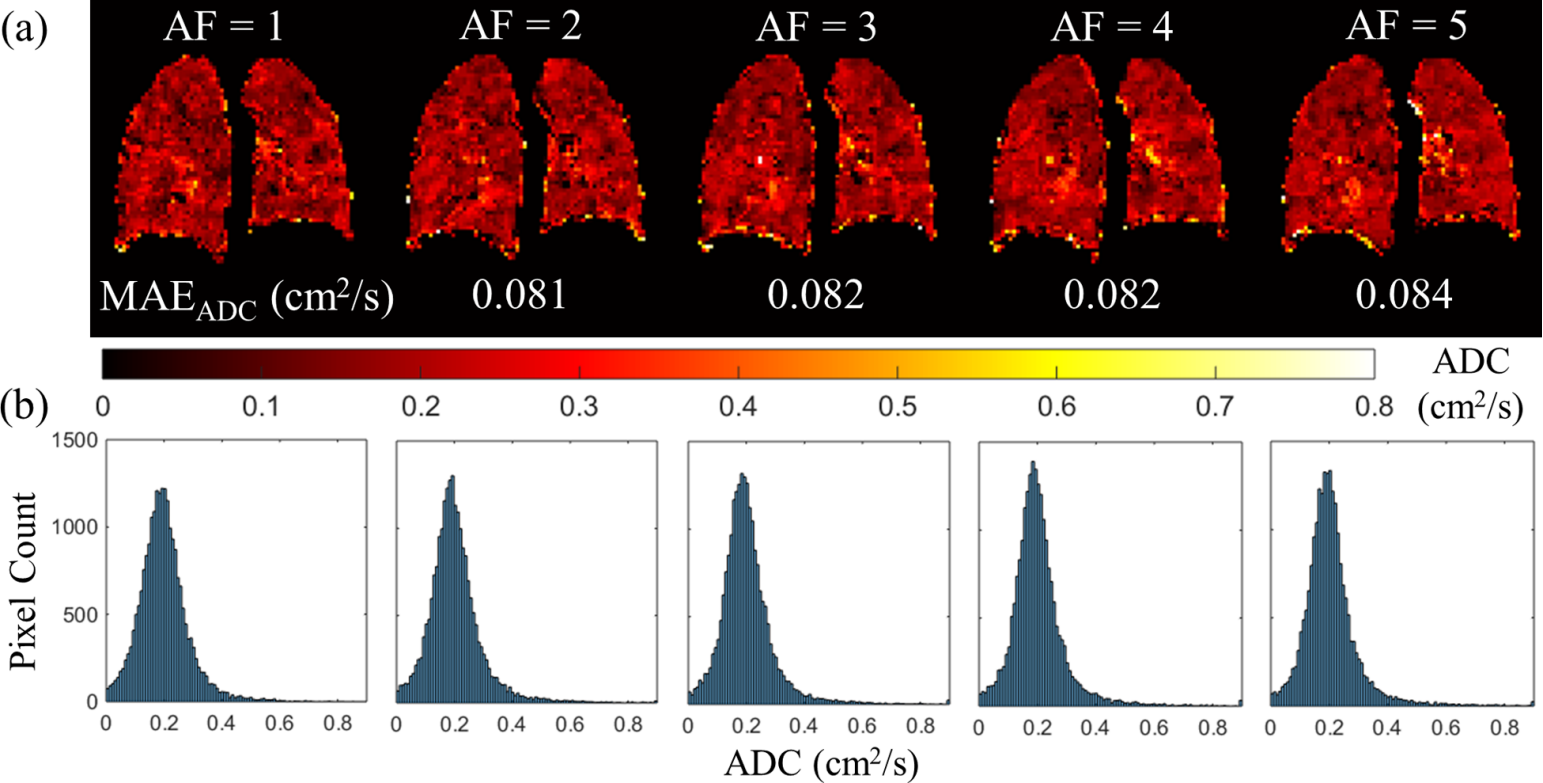
ADC results from 3D ^3^He CS simulations. (a) ADC maps for an example slice at each AF, with corresponding MAE_ADC_ values. (b) Whole lung ADC histograms for each AF.

ADC maps were computed for each reconstructed 3D ^3^He CS dataset and compared with the 3D fully‐sampled ADC maps. CS simulation ADC results are summarized in Table 1 and Figure 2. MAE_ADC_ exhibited a similar trend to MAE_MAG_, ie, increasing undersampling resulted in larger error values. The skewness of the whole lung ADC histograms increased with AF; skewness = 1.08 at AF = 1 and 2.14 at AF = 5, respectively. The opposite trend was observed with the FWHM of the histogram, which decreased at higher AFs: FWHM = 0.141 cm^2^/s at AF = 1 and 0.118 cm^2^/s at AF = 5. In addition, a slight increase in global ADC values was observed with increasing AF, reflecting the increase in MAE_ADC_. The maximum difference in global ADC value between CS and fully‐sampled acquisitions was 4% at AF = 5. Despite the slight increase in global ADC values, single‐slice ADC maps and whole lung ADC histograms for each AF (see Figs. 2a and 2b) appeared to be visually similar, indicating good preservation of quantitative lung microstructural information.

**Table 1:** Summary of Global ADC Values and Whole Lung ADC Histogram Results From 3D ^3^He CS Simulations.

AF	ADC_Global_ (cm^2^/s)	Skewness	FWHM (cm^2^/s)
1^a^	0.198 ± 0.085	1.08	0.141
2	0.203 ± 0.094	1.79	0.127
3	0.202 ± 0.091	1.95	0.123
4	0.204 ± 0.093	2.12	0.120
5	0.206 ± 0.094	2.14	0.118

ADC_Global_, global ADC; FWHM, full width at half maximum

a AF = 1 corresponds to the fully‐sampled dataset.

### Prospective CS Acquisition of 3D ^3^He DW‐MRI

**Figure 3:**
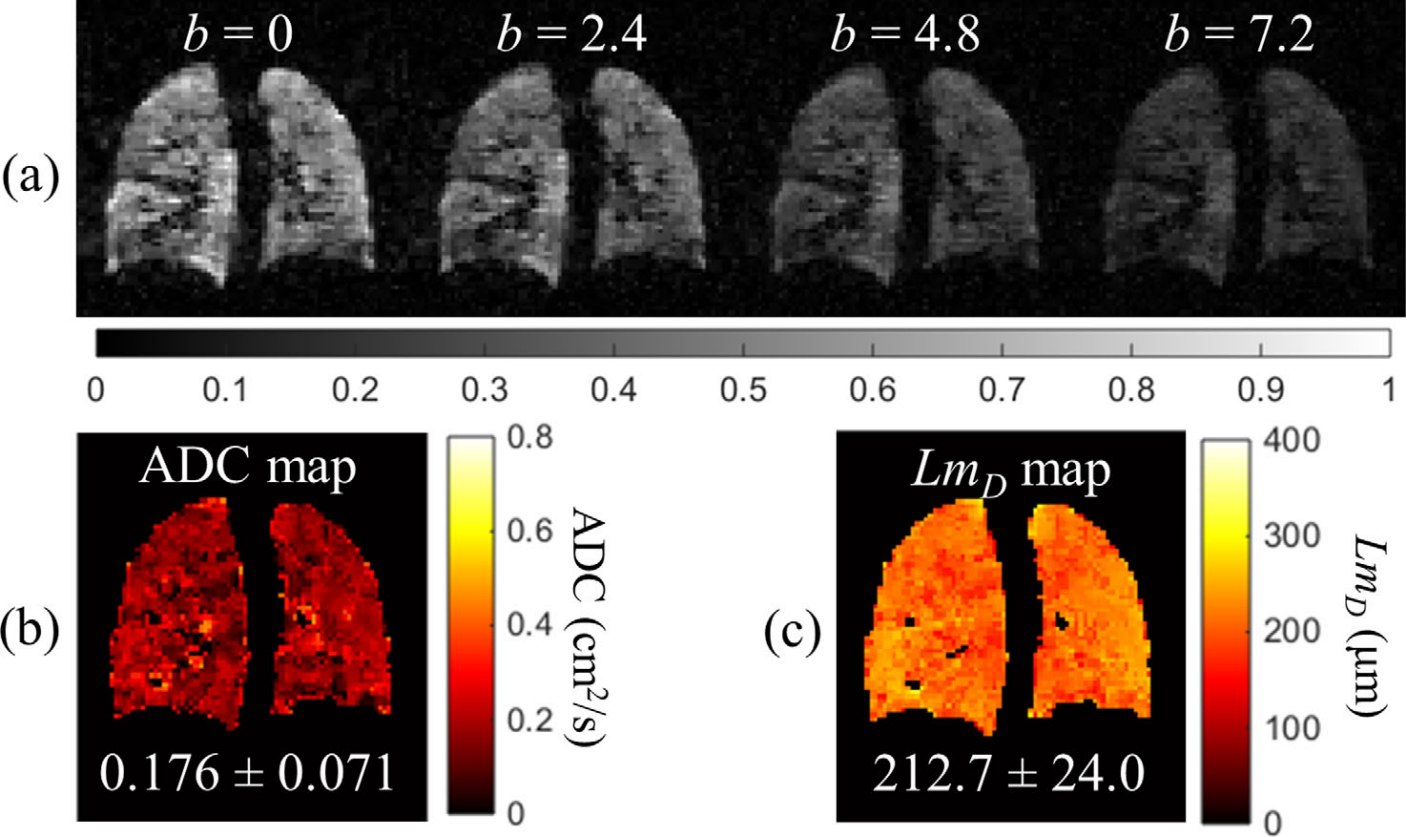
Prospective 3D ^3^He DW‐MRI CS acquisition from a healthy volunteer with three‐fold undersampling and four diffusion interleaves at *b*‐values of 0, 1.6, 4.2, and 7.2 s/cm^2^ (15 s breath‐hold). (a) Example slice for each diffusion interleave. (b) ADC map of the same slice calculated from the first (*b* = 0) and second (*b* = 1.6 s/cm^2^) interleaves. The representative mean slice ADC value is shown underneath the map. (c) Mean alveolar dimension (*Lm*_*D*_) map for the same slice. The representative mean slice *Lm*_*D*_ value is quoted underneath the map.

**Figure 4:**
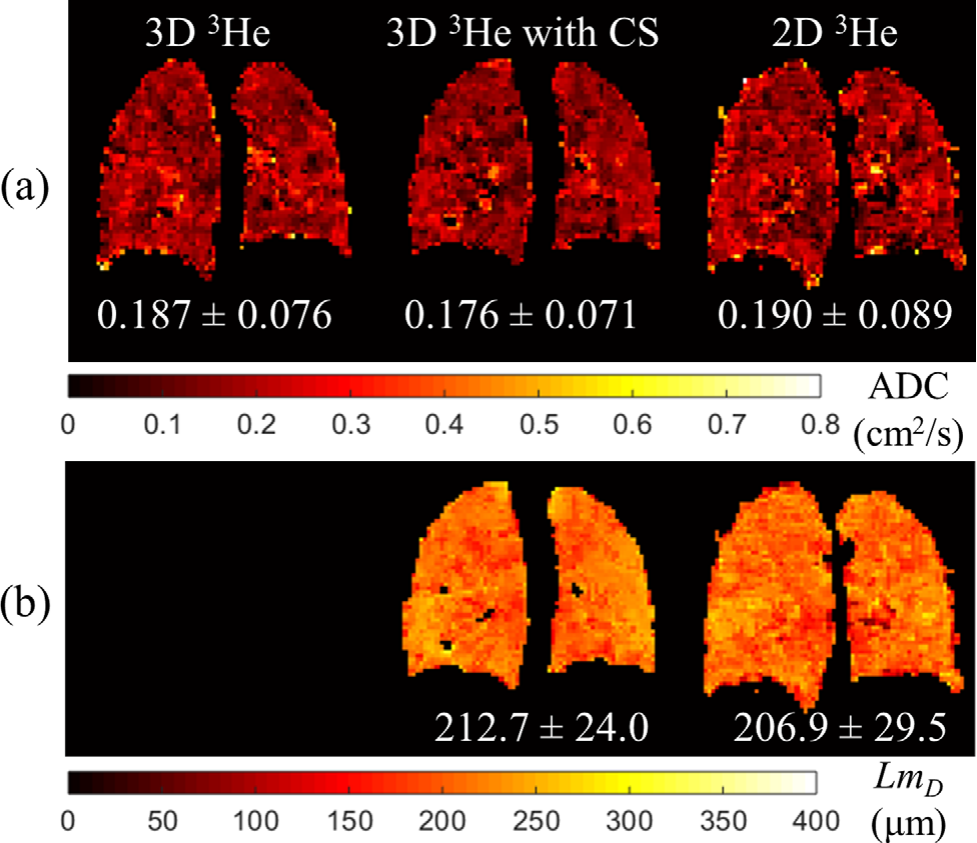
Comparison of 3D and 2D ^3^He ADC and *Lm*_*D*_ maps. (a) Same representative slice ADC map for each of the three imaging methods (3D fully‐sampled, 3D with CS, and 2D fully‐sampled). (b) Corresponding *Lm*_*D*_ maps for 3D with CS and 2D acquisitions.

The four‐interleaved 3D ^3^He DW‐MRI dataset acquired from the same healthy volunteer as previously was reconstructed from the three‐fold undersampled k‐space using the optimal reconstruction parameters of the corresponding undersampling pattern, as determined from CS simulations. Results are summarized in Figures 3 and 4, and Table 2, and described subsequently. A decrease in signal intensity with increasing *b*‐value was observed, corresponding to the increased signal dephasing in the presence of larger diffusion gradients (Fig. 3a). The first two interleaves (*b* = 0 and 1.6 s/cm^2^) were used to calculate an ADC map (Fig. 3b), which resulted in a mean global (whole lung) ADC value of 0.198 ± 0.082 cm^2^/s. All four interleaves of this prospective dataset were then used to generate a *Lm*_*D*_ map from the stretched exponential model (Fig. 3c), which resulted in a mean global *Lm*_*D*_ value of 222.8 ± 25.3 μm.

**Table 2:** Summary of Global and Representative Slice Mean ADC and *Lm*_*D*_ Values Acquired From a Single Healthy Volunteer.

Imaging method	*b*‐value = [0, 1.6 s/cm^2^]		*b*‐value = [0, 1.6, 4.2, 7.2 s/cm^2^]	
	Slice ADC mean (cm^2^/s)	Global ADC mean (cm^2^/s)	Slice *Lm*_*D*_ mean (μm)	Global *Lm*_*D*_ mean (μm)
3D full k‐space	0.187 ± 0.076	0.198 ± 0.085	—a	—a
3D CS	0.176 ± 0.071	0.198 ± 0.082	212.7 ± 24.0	222.8 ± 25.3
2D full k‐space	0.190 ± 0.089	0.194 ± 0.090	206.9 ± 29.5	208.6 ± 29.8

a 3D fully‐sampled data were acquired with only two diffusion interleaves; therefore, no *Lm*_*D*_ value was calculated.

The resulting global and representative slice mean ADC and *Lm*_*D*_ values for this healthy volunteer calculated from 3D CS, 3D fully‐sampled, and 2D fully‐sampled acquisitions are summarized in Table 2. Derived 3D fully‐sampled and 3D CS global ADC values were identical, with a comparable standard deviation, whereas a difference of 6.8% was observed in the global *Lm*_*D*_ value between the 3D CS and the 2D fully‐sampled datasets. In the chosen example slices, a difference in mean ADC of 5.9% between 3D CS and 3D fully‐sampled datasets was observed, as illustrated in Figure 4. For mean LmD, the difference was 2.8% between 3D CS and 2D fully‐sampled datasets.

### Prospective CS Acquisition Validation

A summary of global mean ADC and *Lm*_*D*_ values for all subjects imaged (five healthy volunteers and one COPD patient) is presented in Table 3. For every subject, the global mean ADC value derived from the 3D CS acquisition was equal to or higher than the value obtained from the corresponding 3D fully‐sampled acquisition. The difference in ADC values between the fully‐sampled and CS datasets ranged from 0.0 to 5.9% with a mean difference of 3.4%. Global *Lm*_*D*_ values exhibited a similar increase in 3D CS acquisitions, and a slightly higher mean difference of 5.1% was observed.

**Table 3:** Global ADC and *Lm*_*D*_ Values Calculated From Fully‐Sampled and CS Acquisitions for the Five Healthy Volunteers and One COPD Patient.

Subject	Imaging method	*b*‐value = [0, 1.6 s/cm^2^]	ADC % difference	Multiple *b*‐values	*Lm*_*D*_ % difference
		global ADC (cm^2^/s)		global *Lm*_*D*_ (μm)	
Healthy 1	Fully‐sampled	0.198 ± 0.085	0.0%	208.6 ± 29.8	6.8%
	3D CS	0.198 ± 0.082		222.8 ± 25.3	
Healthy 2	Fully‐sampled	0.163 ± 0.082	4.3%	192.6 ± 27.0	6.0%
	3D CS	0.170 ± 0.077		204.1 ± 23.2	
Healthy 3	Fully‐sampled	0.152 ± 0.083	5.9%	184.5 ± 31.0	8.3%
	3D CS	0.161 ± 0.069		199.8 ± 27.0	
Healthy 4	Fully‐sampled	0.163 ± 0.068	1.8%	197.5 ± 24.2	3.6%
	3D CS	0.166 ± 0.067		204.6 ± 23.6	
Healthy 5	Fully‐sampled	0.169 ± 0.081	5.9%	207.5 ± 24.6	0.7%
	3D CS	0.179 ± 0.078		209.0 ± 29.1	
COPD 1	Fully‐sampled	0.525 ± 0.169	2.7%	—^a^	—
	3D CS	0.539 ± 0.186		313.6 ± 56.1	

2D fully‐sampled DW‐MRI was not acquired from the COPD patient as a result of time constraints.

**Figure 5:**
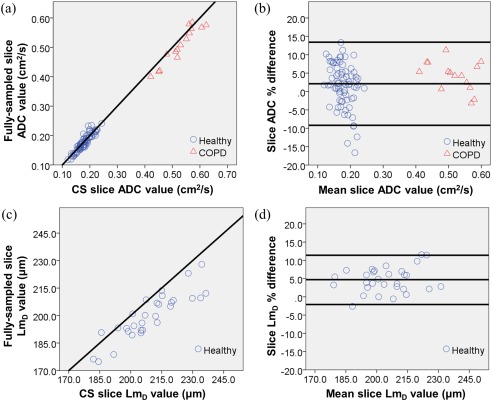
(a) Slice‐by‐slice comparison of mean ADC values between 3D fully‐sampled and 3D CS datasets for all five healthy volunteers and one COPD patient. Solid line represents the line of equality. (b) Bland‐Altman plot showing the agreement between the two methods. The percentage difference in slice ADC values is plotted against the mean slice ADC values between the two measurements. Solid lines represent the mean percentage difference, and the 95% difference range (±1.96 standard deviations). (c) Equivalent slice‐by‐slice comparison of mean *Lm*_*D*_ values between 2D fully‐sampled and 3D CS datasets for all five healthy volunteers. (d) Equivalent *Lm*_*D*_ Bland‐Altman plot showing a similar agreement between the two methods.

A scatter plot of single‐slice ADC values calculated from 3D fully‐sampled and 3D CS datasets (Fig. 5a) shows a good correlation (*P* < 0.001, r = 0.995). Two clusters of data points were observed, corresponding to the healthy and COPD patient groups. The agreement between the two measurements was confirmed by Bland‐Altman analysis (Fig. 5b). The mean slice‐by‐slice ADC percentage difference between methods was +2.1% (absolute difference of 0.005 cm^2^/s), and 95% of the difference was within −9.2% to +13.4% (−0.024 to 0.034 cm^2^/s). Similar levels of agreement in the *Lm*_*D*_ value between methods were observed, as illustrated in the equivalent scatter and Bland‐Altman plots (Figs. 5c and 5d). The mean *Lm*_*D*_ percentage difference of +4.7% (absolute difference of 9.29 μm) was larger than the mean ADC percentage difference, and 95% of the difference was within −2.1% to +11.4% (−4.65 to 23.23 μm).

## DISCUSSION

### CS Simulations

CS has enabled the acquisition of 3D multiple *b*‐value DW lung images with HP ^3^He in a single breath‐hold, for the purpose of diffusion modeling of lung microstructure using a stretched exponential approach. CS simulations performed on a fully‐sampled two *b*‐value 3D ^3^He DW‐MRI dataset led to optimal sampling patterns and penalty weight parameters for different AFs (between 2 and 5). The slight increase in mean ADC value observed with retrospective undersampling was within the range of healthy lung ADC values (∼0.20 cm^2^/s) at *b* = 1.6 s/cm^2^ found in previous studies 2, 3, 24.

Skewness of the ADC histogram was observed to increase with AF. The increase in skewness and mean ADC can both be explained by the increase in mean absolute error (MAE_MAG_ and MAE_ADC_) with increased undersampling. With CS, some information loss is inevitable from the undersampling of k‐space, which increases errors and creates a noise‐like artifact in the magnitude images. The slight increase in MAE_ADC_ results in a few physiologically unrealistic low and high ADC values in some pixels. Examples can be observed in the single‐slice ADC maps at AFs of 2–5 in Figure 2a. An asymmetric cutoff of negative (ADC < 0), or physically too high (ADC > D_0_ = 0.88) values is applied during the creation of the ADC maps; however, some of the uncut artificially high pixel values still influence the mean and skewness of the histogram. If the whole histogram is considered (ie, no cutoff of lower and higher ADC limits is applied), the change in skewness of the ADC histogram between AFs is much lower and the maximum difference in global ADC value becomes only 1.3%, compared with the 4% difference calculated when the asymmetric cutoff is applied.

ADC histograms also appear narrower at larger AFs because of the smaller FWHM values observed with increased undersampling. This trend can be explained by the properties of CS reconstruction, in that denoising and thresholding is used to remove undersampling noise artifacts introduced by undersampling 13. Narrower ADC histograms from increasingly undersampled 2D ^3^He ADC data were also observed previously in 14. The standard deviation values of the global and slice ADC values from reconstructed CS datasets were larger than the corresponding fully‐sampled values; a trend opposite to that reported by Ajraoui et al 14. This difference is likely the result of pixels with high ADC value being introduced from increased MAE_ADC_, causing a larger standard deviation of ADC values. The decrease in FWHM value observed with increased undersampling more accurately reflects the denoising and smoothing of the CS reconstruction process.

In this work, CS simulations were optimized by minimizing MAE_ADC_, whereas in previous work, only MAE_MAG_ was minimized for 2D ADC mapping with ^3^He 14. Here, the ADC values obtained from simulations optimized with minimum MAE_ADC_ were found to be more comparable with fully‐sampled datasets than those optimized using the minimum MAE_MAG_ method. The optimal penalty weight parameters ( $\lambda_{1}$ and $\lambda_{2}$) for simulations with minimum MAE_ADC_ were also found to be smaller than those resulting from MAE_MAG_ simulations. Considering the nonlinear CS reconstruction algorithm in Eq. (1), this finding indicates that the reconstructed images with minimum MAE_ADC_ have less total variation and sparsity when compared with images reconstructed with minimum MAE_MAG_, implying that data consistency needs to be better maintained during the reconstruction process, leading to improved preservation of quantitative lung microstructural information.

As expected, MAE_MAG_ increased with AF in CS simulations, which was manifested as a blurring in image detail due to a reduction in sampling of high‐frequency k‐space components at higher AFs. These blurring effects are an intrinsic result of the variable density k‐space sampling patterns used in the CS simulations. Because most of the information in HP gas lung images arises from low‐frequency k‐space components, a probability density function is used to sample the center of k‐space more heavily than the periphery. In this work, the increasingly lower sampling density of high‐frequency components with increasing AF prevented the use of AFs of 4 and 5 for 3D ^3^He lung MRI acquisitions, because the associated loss of spatial resolution of the reconstructed images was considerable.

### Prospective CS Acquisition of 3D DW‐MRI

A prospective three‐fold undersampled 3D ^3^He DW‐MRI dataset was acquired in one healthy volunteer using an optimized undersampling pattern, and quantitative measures of lung microstructure were compared with 2D fully‐sampled and 3D DW‐MRI datasets. An excellent ADC agreement was observed between 3D fully‐sampled and 3D CS datasets, whereas a difference of 6.8% was observed in the global *Lm*_*D*_ value between the 3D CS and 2D fully‐sampled datasets. This global *Lm*_*D*_ difference was within the standard deviation range of the global mean value, and *Lm*_*D*_ values were similar to reported mean linear intercept values obtained from healthy human lung histology samples (∼200 μm) 25. In the chosen example slices, the observed mismatch between the CS and fully‐sampled ADC and *Lm*_*D*_ values could be explained by slight differences in subject position or lung inflation level between the separate scans, which could cause the example slices to be representative of a slightly different region of the lungs (Fig. 4).

A small positive bias in global mean ADC and *Lm*_*D*_ value was observed between 3D CS and fully‐sampled 2D and 3D datasets acquired from the five healthy volunteers and one COPD patient, which could be attributed to the increase in MAE_ADC_ as a result of undersampling. However, these values were within the standard deviation range, consistent with reported healthy and COPD lung ADC values 2, 3, 24 and similar to mean linear intercept values obtained from human lung histology samples (∼200 μm in healthy, ∼400 μm in COPD) 25. In a slice‐by‐slice comparison of fully‐sampled and CS‐derived ADC and *Lm*_*D*_ values, good agreement was found, close to the line of equality. In the quantitative comparisons of both ADC and *Lm*_*D*_, 95% of the difference between fully‐sampled and CS datasets was well within the standard deviation range of mean values. From the CS simulations (where, unlike the fully‐sampled acquisitions, there is intrinsically no variability because of scans being performed in a separate breath), a ∼2% ADC mismatch was observed between the fully‐sampled and three‐fold undersampled CS reconstruction, which can be attributed to CS reconstruction error.

Despite the observation of a small positive bias in CS‐derived ADC and *Lm*_*D*_ values, the biases are negligible when compared with the large differences in lung microstructure between healthy and COPD subjects; ADC and mean linear intercept length values of emphysema subjects vary depending on disease severity, but are typically 2–2.5 times larger than those of healthy subjects 2, 25. Thus, our results indicate that 3D multiple *b*‐value ^3^He DW‐MRI with CS has potential to be used clinically to track changes in lung microstructure associated with emphysematous disease. The 3D multiple *b*‐value data affords the possibility of calculating *Lm*_*D*_ across the entire lung volume from the stretched exponential model, allowing for volumetric lung microstructural estimates. The 3D multiple *b*‐value acquisition strategy proposed here is fully compatible with the stretched exponential model, and also alternative diffusion analyses and morphometric models, such as the “cylinder model” 5, 6 or q‐space transform analysis 7.

**Figure 6:**
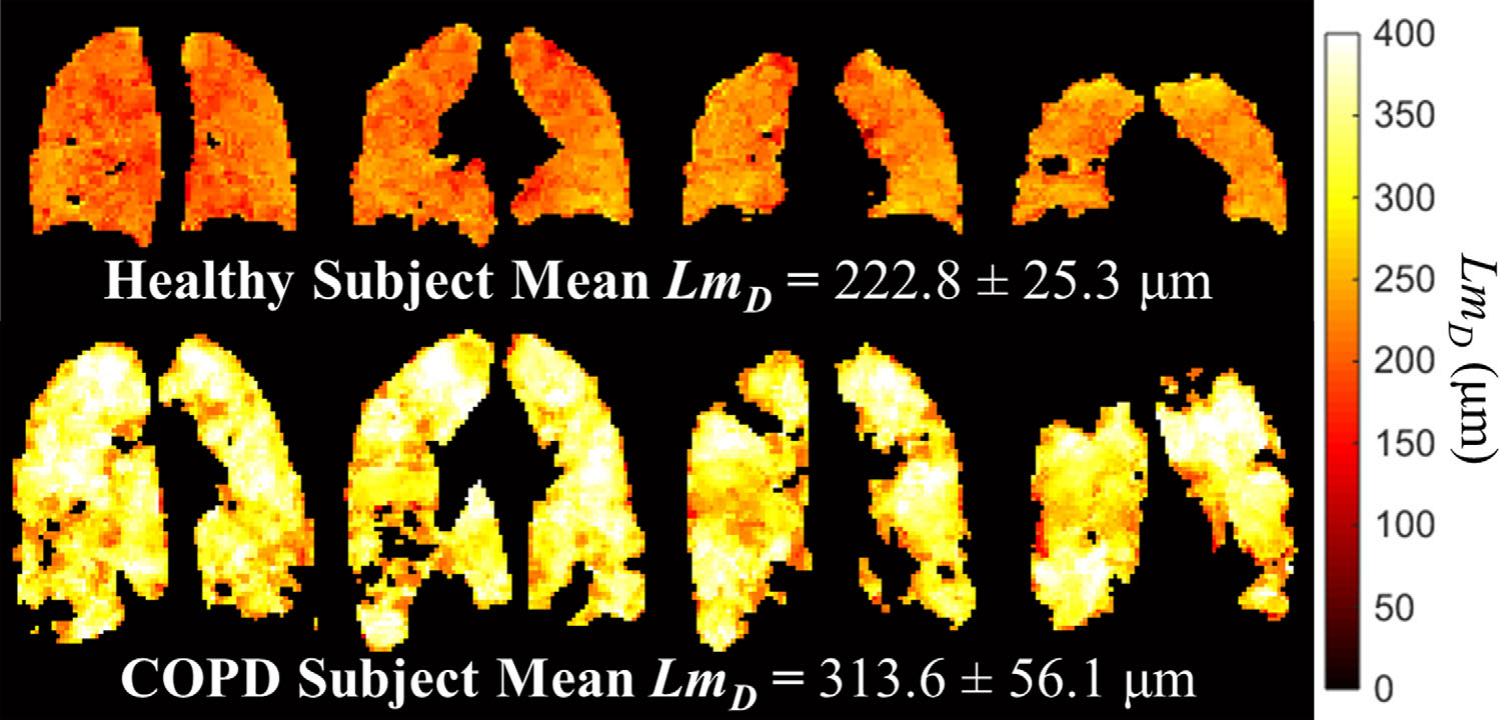
Four example slice *Lm*_*D*_ maps calculated from 3D multiple *b*‐value ^3^He DW‐MRI with CS acquired in a healthy (top row) and COPD subject (bottom row).

To demonstrate the clinical potential of this method, five healthy subjects and one COPD patient were imaged with the 3D CS multiple *b*‐value DW‐MRI sequence. *Lm*_*D*_ maps were calculated across the entire lung for each subject, and the derived global mean *Lm*_*D*_ values reflect the alveolar size of each subject (example maps from a healthy subject and the COPD patient are shown in Fig. 6).

One limitation of the slice‐by‐slice comparison of lung microstructural measurements between fully‐sampled and CS datasets was that the lung volume was assumed to be the same for each acquisition. Subjects were instructed to inhale the ^3^He and N_2_ gas dosage from FRC to produce a lung volume of FRC + 1 L. However, this inflation volume may not have been reproduced exactly for each acquisition. A difference in lung inflation level of 6% compared with 15% of total lung capacity (TLC) has been shown to have a relatively minor effect on the global mean ADC values 24, but this could also result in slices from the fully‐sampled and CS datasets that correspond to slightly different regions of the lung. The difference in lung microstructural parameters between possibly misregistered slices could be additionally affected by the gravitational dependence of lung ventilation. For example, in the supine position, ^3^He ADC values have been shown to be larger in the anterior regions of the lung compared with the posterior regions, as a result of the gravitational compression of lung tissue in the latter 26.

From the CS simulations, a small increase in MAE_ADC_ and MAE_MAG_ was observed from AF = 3 to AF = 4 or 5. The use of these high AFs could allow for higher nominal spatial resolutions to be achieved, or permit the acquisition of additional diffusion interleaves. However, one drawback of using higher AFs is the more severe image blurring effects introduced as a result of the heavier undersampling of high‐frequency k‐space components. These blurring effects could be reduced by incorporating prior knowledge into the CS reconstruction procedure to improve reconstruction accuracy 15, and thus improve the preservation of edge details of the 3D CS multiple *b*‐value DW‐MRI lung images.

In recent years, the potential of HP ^129^Xe as a cost‐effective alternative to ^3^He for lung imaging and ADC mapping has been explored, with comparable results and functional information obtained from the two nuclei 1, 27. Furthermore, naturally abundant xenon (26% ^129^Xe) 28 and efficient use of enriched xenon 29 have been shown to enable high‐quality ventilation imaging at a significantly lower cost than that of ^3^He. However, the approximately three‐fold lower gyromagnetic ratio of ^129^Xe compared with ^3^He translates to a considerable signal disadvantage under equivalent experimental conditions, and the lower diffusivity of the xenon gas also requires that longer diffusion gradients be used to probe lung microstructure in DW‐MRI. To date, 2D multiple *b*‐value ^129^Xe DW‐MRI has been demonstrated for lung morphometry assessment 30, 31; however, these methods do not provide whole lung coverage information. The CS techniques implemented here are readily translatable to ^129^Xe and could be applied to enable acquisition of 3D multiple *b*‐value ^129^Xe DW‐MRI, to allow whole lung morphometry calculations at a fraction of the cost of an equivalent ^3^He acquisition.

## CONCLUSIONS

Compressed sensing has been implemented successfully for the acquisition of 3D multiple *b*‐value DW‐MRI lung images with HP ^3^He in a single breath‐hold for quantitative whole lung microstructural assessment. Prospective CS datasets were acquired in five healthy volunteers and one COPD patient using an optimized three‐fold undersampled k‐space pattern, and derived ADC and *Lm*_*D*_ results were validated against fully‐sampled 3D and 2D ^3^He DW‐MRI. Good agreement between prospective CS and fully‐sampled datasets was found, with a mean difference of +3.4 and +5.1% in global mean ADC and *Lm*_*D*_ values, respectively. These results confirm that CS acquisition of undersampled 3D ^3^He MRI datasets with multiple *b*‐values for lung morphometry is fit for clinical lung imaging studies.
